# Cas13b-mediated RNA targeted therapy alleviates genetic dilated cardiomyopathy in mice

**DOI:** 10.1186/s13578-023-01143-y

**Published:** 2024-01-04

**Authors:** Jiacheng Li, He Xuan, Xin Kuang, Yahuan Li, Hong Lian, Nie Yu

**Affiliations:** 1 State Key Laboratory of Cardiovascular Disease, Fuwai Hospital, National Center for Cardiovascular Disease, Chinese Academy of Medical Sciences and Peking Union Medical College, Beijing, 100037 China; 2 National Health Commission Key Laboratory of Cardiovascular Regenerative Medicine, Fuwai Central-China Hospital, Central-China Branch of National Center for Cardiovascular Diseases, Zhengzhou, 450046 China; 3 Center for Reproductive Medicine, Department of Obstetrics and Gynecology, Peking University Third Hospital, Beijing, 100191 China; 4 National Clinical Research Center for Obstetrics and Gynecology, Peking University Third Hospital), Beijing, 100191 China; 5 Key Laboratory of Assisted Reproduction (Peking University), Ministry of Education, Beijing, 100191 China; 6 Beijing Key Laboratory of Reproductive Endocrinology and Assisted Reproductive Technology, Beijing, 100191 China

**Keywords:** PspCas13b, Cardiac troponin T, Dilated cardimyopathy, Gene therapy

## Abstract

**Background:**

Recent advances in gene editing technology have opened up new avenues for in vivo gene therapy, which holds great promise as a potential treatment method for dilated cardiomyopathy (DCM). The CRISPR-Cas13 system has been shown to be an effective tool for knocking down RNA expression in mammalian cells. PspCas13b, a type VI-B effector that can be packed into adeno-associated viruses and improve RNA knockdown efficiency, is a potential treatment for diseases characterized by abnormal gene expression.

**Results:**

Using PspCas13b, we were able to efficiently and specifically knockdown the mutant transcripts in the AC16 cell line carrying the heterozygous human *TNNT2*^*R141W*^ (*hTNNT2*^*R141W*^) mutation. We used adeno-associated virus vector serotype 9 to deliver PspCas13b with specific single guide RNA into the *hTNNT2*^*R141W*^ transgenic DCM mouse model, effectively knocking down *hTNNT2*^*R141W*^ transcript expression. PspCas13b-mediated knockdown significantly increased myofilament sensitivity to Ca^2+^, improved cardiac function, and reduced myocardial fibrosis in *hTNNT2*^*R141W*^ DCM mice.

**Conclusions:**

These findings suggest that targeting genes through Cas13b is a promising approach for in vivo gene therapy for genetic diseases caused by aberrant gene expression. Our study provides further evidence of Cas13b’s application in genetic disease therapy and paves the way for future applicability of genetic therapies for cardiomyopathy.

## Background

RNA-based therapeutics have been investigated in the past decade for the treatment of various diseases, such as cardiovascular disorders [1, 2], cancer [3–5], central nervous system disease [6–8] and endocrine system disease [9–11]. Conventional methods targeting transcripts using RNA interference (RNAi) or antisense oligonucleotides have significant nonspecific and off-target effects [12, 13]. However, a member of a family of RNA-targeting CRISPR effectors called Cas13 has demonstrated a robust ability to downregulate cellular RNAs in mammalian cells in vitro [14–17]. Cas13b represents a significant improvement over existing RNA-targeting methods due to its lower off-target effects, with the PspCas13b subtype displaying the most potent RNA knockdown efficiency [18]. Furthermore, PspCas13b is a type VI-B effector with a small size that can be packaged into adeno-associated viruses (AAVs), making it a promising therapeutic tool for diseases characterized by aberrant gene expression.

Dilated cardiomyopathy (DCM) is a cardiac disorder characterized by dilatation and impaired contraction of the left ventricle or both ventricles and is a leading cause of heart failure and heart transplantation [19–21]. Familial DCM accounts for 20–48% of cases, a large proportion of which are principally results of mutations in the genes encoding sarcomeric proteins in cardiomyocytes [22, 23]. Troponin T, encoded by the *TNNT2* gene, is one such sarcomeric protein. Troponin T binds troponin C and troponin I to form the troponin complex that regulates muscle contraction via calcium sensitivity through conformational changes in the interaction between cardiac actin and myosin heavy chain [24, 25]. The *TNNT2*^*R141W*^ variant was first discovered in a five-generation DCM family using the combination of Sanger sequencing of candidate genes and restriction-endonuclease analysis [24]. To investigate the functional consequences of this variant, Zhang et al. [26] generated a humanized *TNNT2*^*R141W*^ transgenic mouse model (R141W mice), which exhibits a progressive and severe form of DCM by the age of 4 months. Numerous studies have delved into the pathological mechanisms pertaining to the human *TNNT2*^*R141W*^ (*hTNNT2*^*R141W*^) mutation in R141W DCM mice [27–30]. Nevertheless, gene therapy research targeting DCM caused by the *TNNT2* mutation needs further study.

Heterozygous mice with one disrupted copy of the *Tnnt2* gene exhibit a mild decrease in transcript expression but no discernable changes in protein expression or phenotype [31]. In addition, current evidence in the ClinVar and DECIPHER databases does not report *TNNT2* haploinsufficiency as a cause of cardiomyopathy. Therefore, we propose that RNA-targeted therapy targeting mutant *TNNT2* mRNA may be a promising approach for treating DCM caused by *TNNT2* mutations.

In this study, we established the PspCas13b system to target *hTNNT2*^*R141W*^ mRNA in a R141W DCM mouse model. We found that adeno-associated virus vector serotype 9 (AAV9)-delivered PspCas13b and single guide RNA (sgRNA) disrupted *hTNNT2*^*R141W*^ mRNA expression and alleviated the cardiac dysfunction in *hTNNT2*^*R141W*^ transgenic mice. These findings are encouraging and will pave the way for future genetic cardiomyopathy therapies.

## Results

### **A* hTNNT2***^***R141W***^** transgenic mouse model with DCM**

**Fig. 1:**
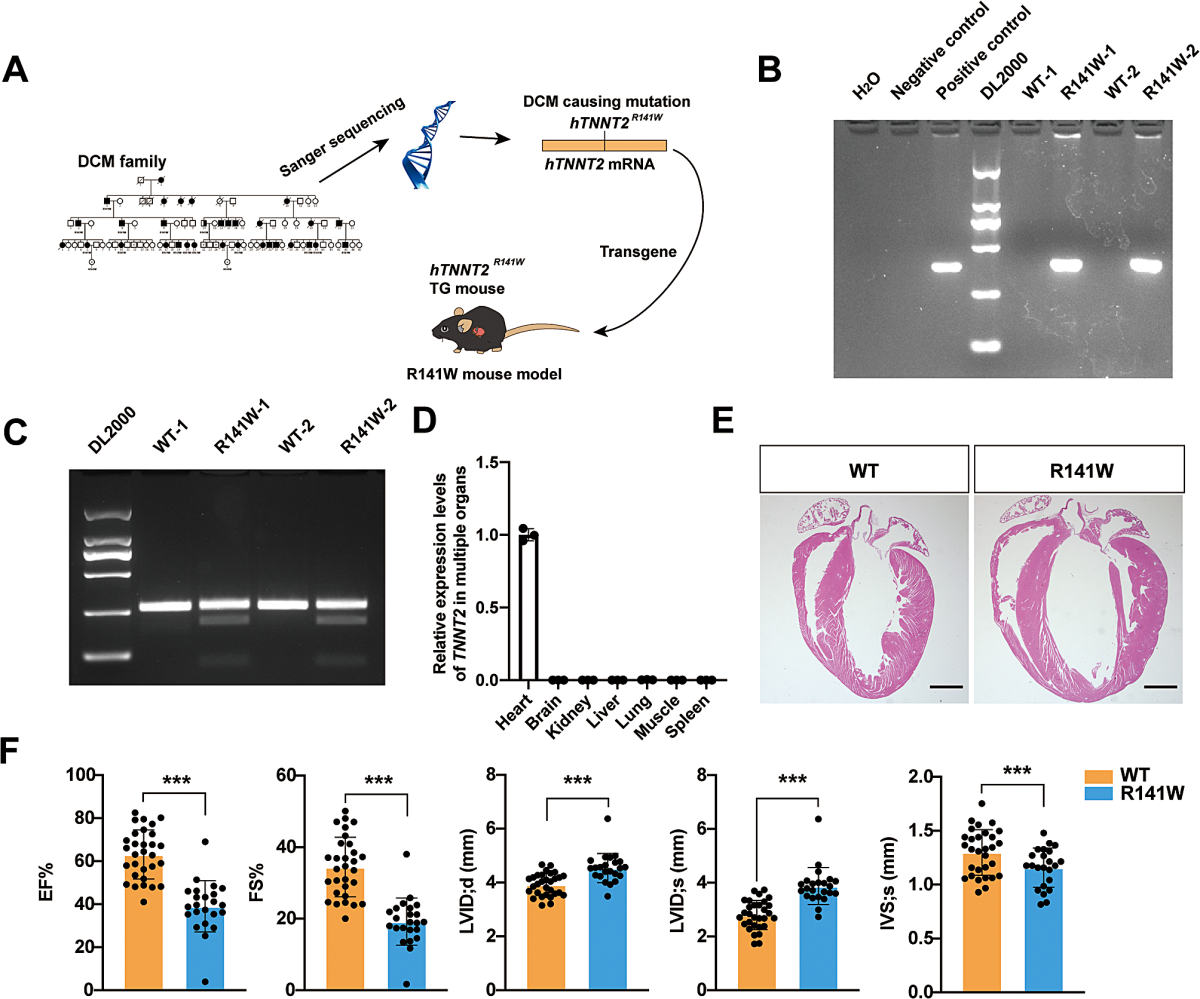
A *hTNNT2*^*R141W*^ transgenic mouse model with DCM. (**A**) Schematic showing the procedure for constructing *hTNNT2*^*R141W*^ transgenic mice (R141W mice). (**B**) DNA gel electrophoresis of PCR-amplified DNA products extracted from mouse tails. The 369 bp PCR products were present in lanes 3, 6 and 8. Lane 1, H_2_O; lane 2, negative control; line 3, positive control; lane 4, DNA molecular weight marker (DL2000); lane 5, WT mouse; lane 6, R141W-1 (*hTNNT2*^*R141W*^ transgenic mice); lane 7, WT mouse; and line 8, R141W-2. (**C**) Undigested and digested *TNNT2* PCR products for WT control (lanes 2 and 4) and R141W mice (lanes 3 and 5). (**D**) qRT-PCR analysis of mRNA levels of *hTNNT2*^*R141W*^ transgene in the heart, liver, spleen, lung and brain. (**E**) Histopathological profile of heart tissue from mice at 4 months of age. H&E staining patterns of whole-heart longitudinal sections are shown. Scale bars = 500 μm. (**F**) Echocardiographic analyses of mouse hearts. Four-month-old WT mice (n = 30) and R141W mice (n = 23) were analyzed by M-mode echocardiography. The transgenic heart showed an enlarged ventricular chamber and decreased movement of the ventricular wall. EF, left ventricular ejection fraction. FS, left ventricular fractional shortening. LVID; d, diastolic left ventricular internal diameter. LVID;s, systolic left ventricular internal diameter. IVS;s, systolic interventricular septum. The bar graphs show the mean ± SEM. ***p < 0.001 according to Student’s t test [32–34]

To determine the mechanistic role of the *hTNNT2*^*R141W*^ variant in DCM, Zhang and colleagues [26] generated a transgenic mouse model expressing the human TNNT2^R141W^ protein, which was maintained on a C57BL/6J genetic background (Fig. 1A). Through genotyping, we amplified a fragment consistent with the positive control in R141W mice (Fig. 1B). By performing HpaII enzyme digestion on the *hTNNT2*^*R141W*^ cDNA amplification product, fragments of 147 bp and 66 bp + 44 bp sizes were cleaved from the R141W mouse cDNA amplicon (Fig. 1C). The expression of *hTNNT2*^*R141W*^ was specifically controlled by the α-MHC promoter in cardiomyocytes. The mRNA levels of *TNNT2* in the heart, liver, spleen, lung, kidney, skeletal muscle, and brain were confirmed by quantitative real-time PCR (qRT-PCR), suggesting that the *hTNNT2*^*R141W*^ gene was specifically expressed in the heart (Fig. 1D). At four months of age, the transgenic mice exhibited ventricular chamber enlargement and systolic dysfunction (Fig. 1E, F). Here we examined the DCM-associated phenotype of the R141W mouse model, implying its potential use for subsequent research.

### **Specific knockdown of* hTNNT2***^***R141W***^** mRNA** in vitro **using PspCas13b**

**Fig. 2:**
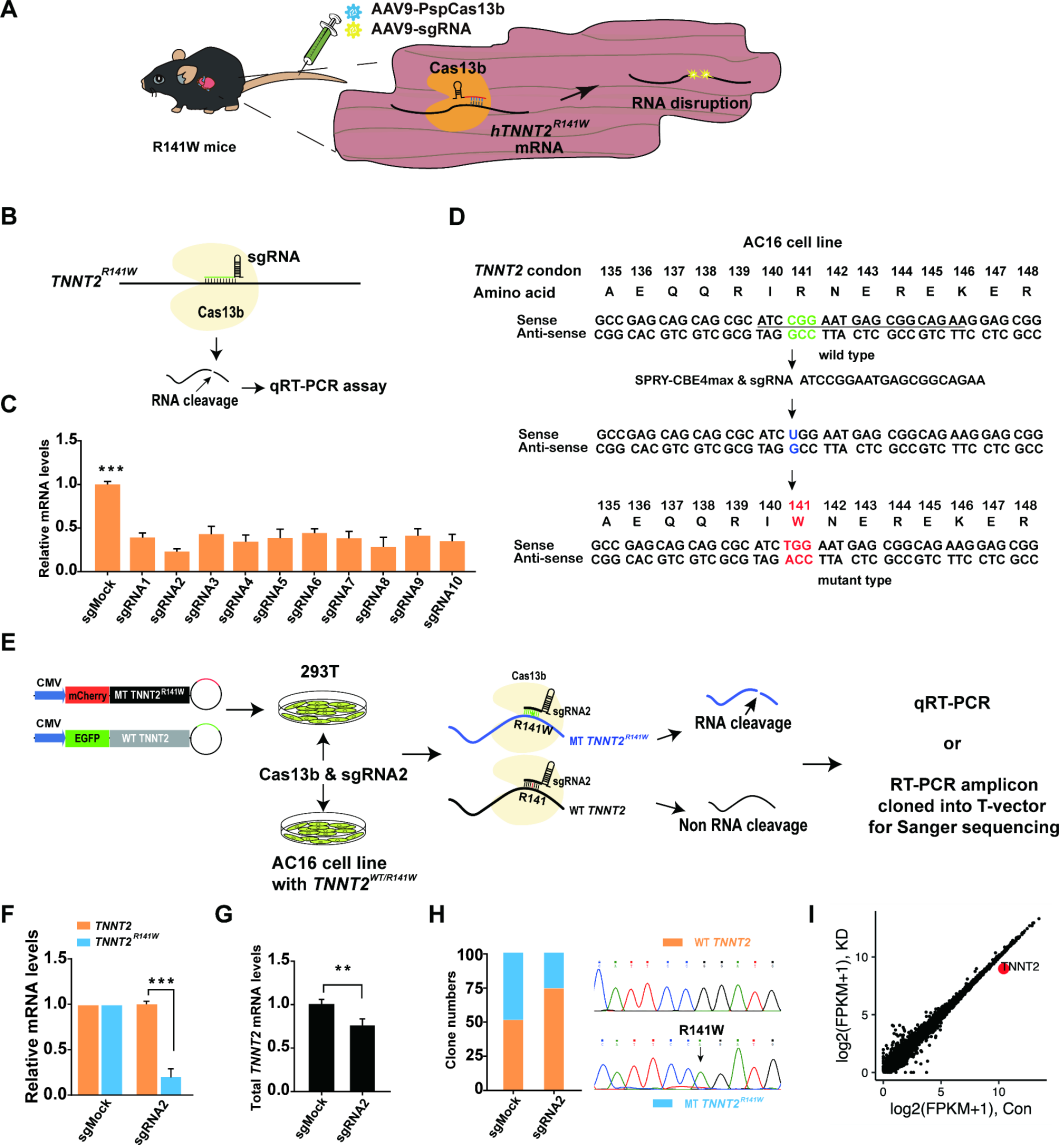
Specific knockdown of *hTNNT2*^*R141W*^ in vitro using PspCas13b. (**A**) Schematic showing the experimental procedure for the delivery of PspCas13b and sg*TNNT2*-expressing AAV9s into *hTNNT2*^*R141W*^ transgenic mice. (**B**) Schematic representation of the mechanism of the PspCas13b-sgRNA system in silencing the expression of the mutant *TNNT2*^*R141W*^ transcript. (**C**) qRT-PCR demonstrating successful knockdown of *hTNNT2*^*R141W*^ in *TNNT2*^*R141W*^-293T cells (n = 4 per group). ***p < 0.001 according to Student’s t test. (**D**) Schematic diagram of the process for constructing an *TNNT2*^*WT/R141W*^-AC16 cell line using SpRY-CBEmax. (**E**) Schematic representation of the process of PspCas13b-specific knockdown of mutant transcript expression in the *TNNT2*^*WT*/*R141W*^-AC16 cell line and the HEK-293T cell line with reporter alleles. (**F**) Assessment of knockdown of WT (green) and mutant (red) mRNA expression in HEK-293T cells transfected with reporter alleles and PspCas13b-sgRNA2 normalized to the levels observed in cells transfected only with the reporter alleles (n = 3 independent experiments). (**G**) qRT-PCR quantification of total *TNNT2* mRNA in *TNNT2*^*WT*/*R141W*^-AC16 cells (n = 6 independent experiments). *p < 0.05 according to Student’s t test. (**H**) Sanger sequencing of T vectors with WT and *hTNNT2*^*R141W*^ transcripts. (**I**) Expression levels in log2 (fragments per kilo base per million mapped reads [FPKM] + 1) values of all detected genes in RNA sequencing (RNA-seq) libraries of PspCas13b-*TNNT2* (y axis) compared to PspCas13b control (x axis) (n = 3 independent replicates for both groups)

In order to effectively knockdown *hTNNT2*^*R141W*^ transcripts for the treatment of DCM, we designed to use AAV9 to deliver PspCas13b along with specific sgRNA into *hTNNT2*^*R141W*^ transgenic mice (Fig. 2A). First, to optimize the PspCas13b-mediated knockdown efficiency of the mutant *TNNT2*^*R141W*^ transcript, we screened for the most effective sgRNAs targeting the *TNNT2* mutant site. Ten *TNNT2*-sgRNAs and PspCas13b vectors were cotransfected into *TNNT2*^*R141W*^ 293T cells that had been infected with EF1α-*TNNT2*^*R141W*^-puro lentivirus (Fig. 2B). Forty-eight hours after transfection, qRT-PCR assays revealed that cotransfection of *TNNT2*-sgRNA2 (sg*TNNT2*) and the PspCas13b vector decreased *TNNT2*^*R141W*^ expression by 75.9 ± 0.97% (Fig. 2C).

To verify the specificity of PspCas13b in targeting *TNNT2*^*R141W*^ mRNA, we inserted wild-type (WT) *TNNT2* (WT-*TNNT2*) and mutant-type *TNNT2* (MT-*TNNT2*^*R141W*^) sequences into EGFP-WT-*TNNT2* and mCherry-MT-*TNNT2*^*R141W*^ vectors, respectively (Fig. 2E). We compared the ability of sg*TNNT2* to knock down MT-*TNNT2*^*R141W*^ mRNA expression relative to WT-*TNNT2* mRNA expression. qRT-PCR data showed that sg*TNNT2* reduced MT-*TNNT2*^*R141W*^ expression by 70% without significantly affecting WT-*TNNT2* expression (Fig. 2F).

To further investigate the specificity of PspCas13b-mediated knockdown of *TNNT2*^*R141W*^, we established an AC16 cell line carrying the heterozygous *TNNT2*^*R141W*^ mutation (Fig. 2D). Cells were infected with PspCas13b and sg*TNNT2* or sgCtrl as a control. Two days after infection, qRT-PCR analysis showed that total *TNNT2* expression was reduced to 72.4 ± 3.1% of that in control cells (Fig. 2E, G). We also ligated the RT-PCR product of the *TNNT2*^*R141W*^ fragment into a T vector and selected 100 colonies for Sanger sequencing. Analysis of the sequencing results revealed that in the sg*TNNT2* group, 79 colonies had a WT sequence, while 21 colonies had a mutant sequence. In contrast, in the control group, 53 colonies had a WT sequence, and 47 colonies had a mutant-type sequence (Fig. 2H). These results suggest that the CRISPR-Cas13b system can efficiently and specifically knock down *TNNT2*^*R141W*^ transcript expression in vitro.

To detect the potential off-target effects, sg*TNNT2*s and PspCas13b vectors were cotransfected into *TNNT2*^*R141W*^ 293T cells that had been infected with EF1α-*TNNT2*^*R141W*^-puro lentivirus. Forty-eight hours after transfection, cells were collected for RNA extraction and subsequent transcriptome-wide mRNA sequencing to detect significant differentially expressed genes. Transcriptome analysis showed that *TNNT2* was specifically downregulated while the transcriptional level of other genes remained unchanged 2 days after transfection (Fig. 2I).

### **PspCas13b effectively knocked down*hTNNT2***^***R141W***^**transcript expression** in vivo

**Fig. 3:**
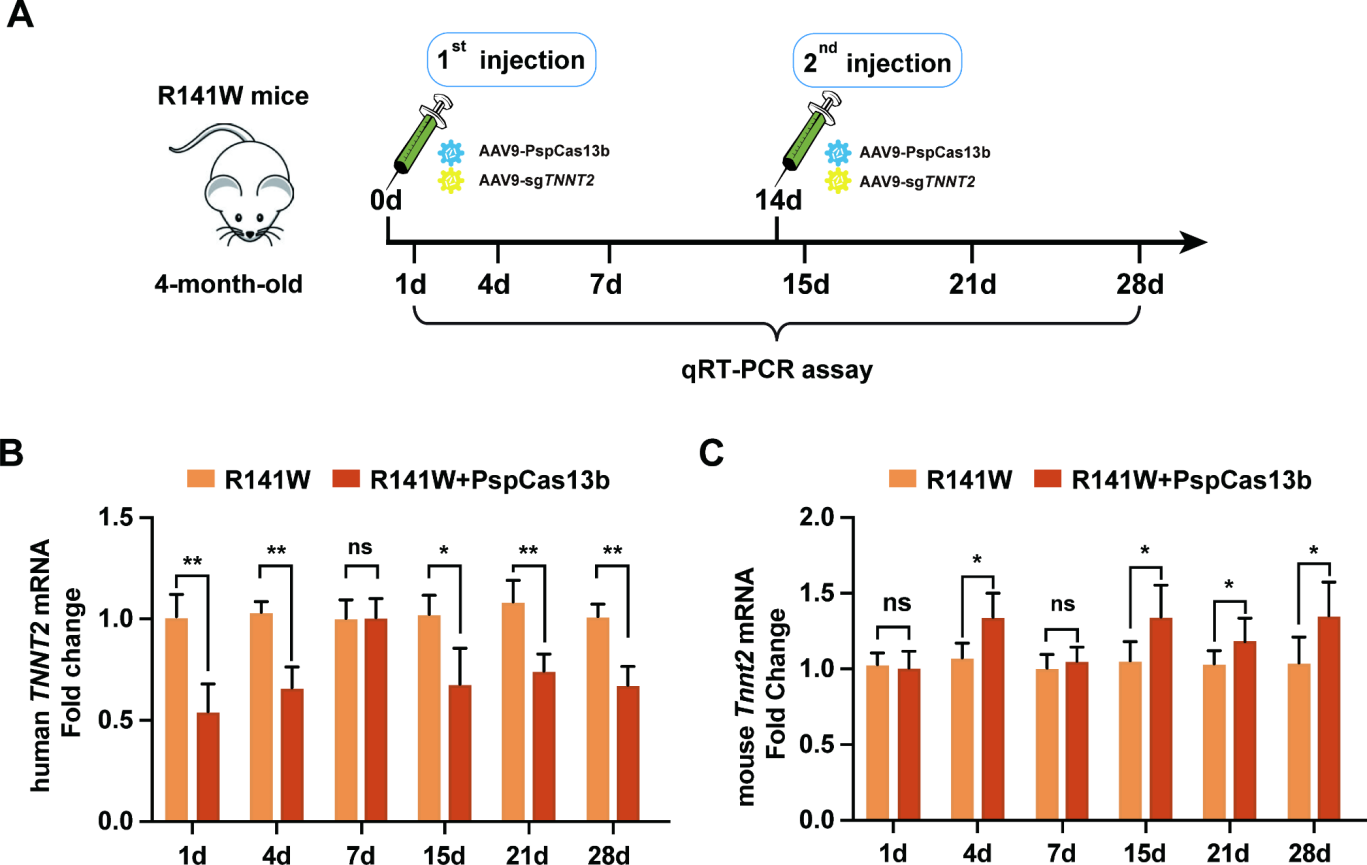
Effective knockdown of *hTNNT2*^R141W^ mRNA expression through PspCas13b in vivo. (**A**) Schematic of the experimental procedure. (**B**) Quantification of *hTNNT2* mRNA levels in hearts from AAV9-PspCas13b-sg*TNNT2*-injected (n = 6) and noninjected R141W mice (n = 3). (**C**) Quantification of the relative expression of the mouse *Tnnt2* gene in DCM and DCM + AAV9 cardiomyocytes by qRT-PCR using the 2^-∆∆Ct^ method (n = 3). The bar graphs show the mean ± SEM. NS is not significant; *p < 0.05 according to Student’s t test

To investigate the feasibility of PspCas13b-mediated knockdown of *hTNNT2*^*R141W*^ mRNA expression in vivo, we injected AAV9s encoding PspCas13b and sg*TNNT2* (AAV9-PspCas13b and AAV9-sg*TNNT2*) into 4-month-old R141W mice via tail vein injection (Fig. 3A). As shown in the schematic diagram, mice received twice tail vein injections, with the second injection administered 14 days after the initial injection. Samples were obtained on days 1, 4, 7, 15, 21, and 28 after the first AAV9 injection to detect *hTNNT2*^*R141W*^ transcript expression levels. On the first day after AAV9-PspCas13b injection, *hTNNT2*^*R141W*^ mRNA levels in cardiomyocytes were 54.4 ± 4.7% of those in cardiomyocytes without AAV9 injection. As PspCas13b vector expression in cardiomyocytes gradually declined, *hTNNT2*^*R141W*^ mRNA levels gradually returned to normal levels by 7 days after injection. The second AAV9-PspCas13b injection achieved similar efficiency of *hTNNT2*^*R141W*^ knockdown as observed in the first injection (Fig. 3B). Furthermore, qRT-PCR analysis revealed significant upregulation of mouse *Tnnt2* (*mTnnt2*) expression on the fourth day after the first injection and at several subsequent time points (Fig. 3C). These results demonstrate that PspCas13b can effectively knock down the expression of the mutant *hTNNT2*^*R141W*^ transcript in vivo. As *hTNNT2*^*R141W*^ expression decreased, *mTnnt2* expression increased to compensate.

### **PspCas13b-mediated*hTNNT2***^***R141W***^**knockdown regulates intracellular Ca**^**2+**^**and sarcomere dynamics in cardiomyocytes**

**Fig. 4:**
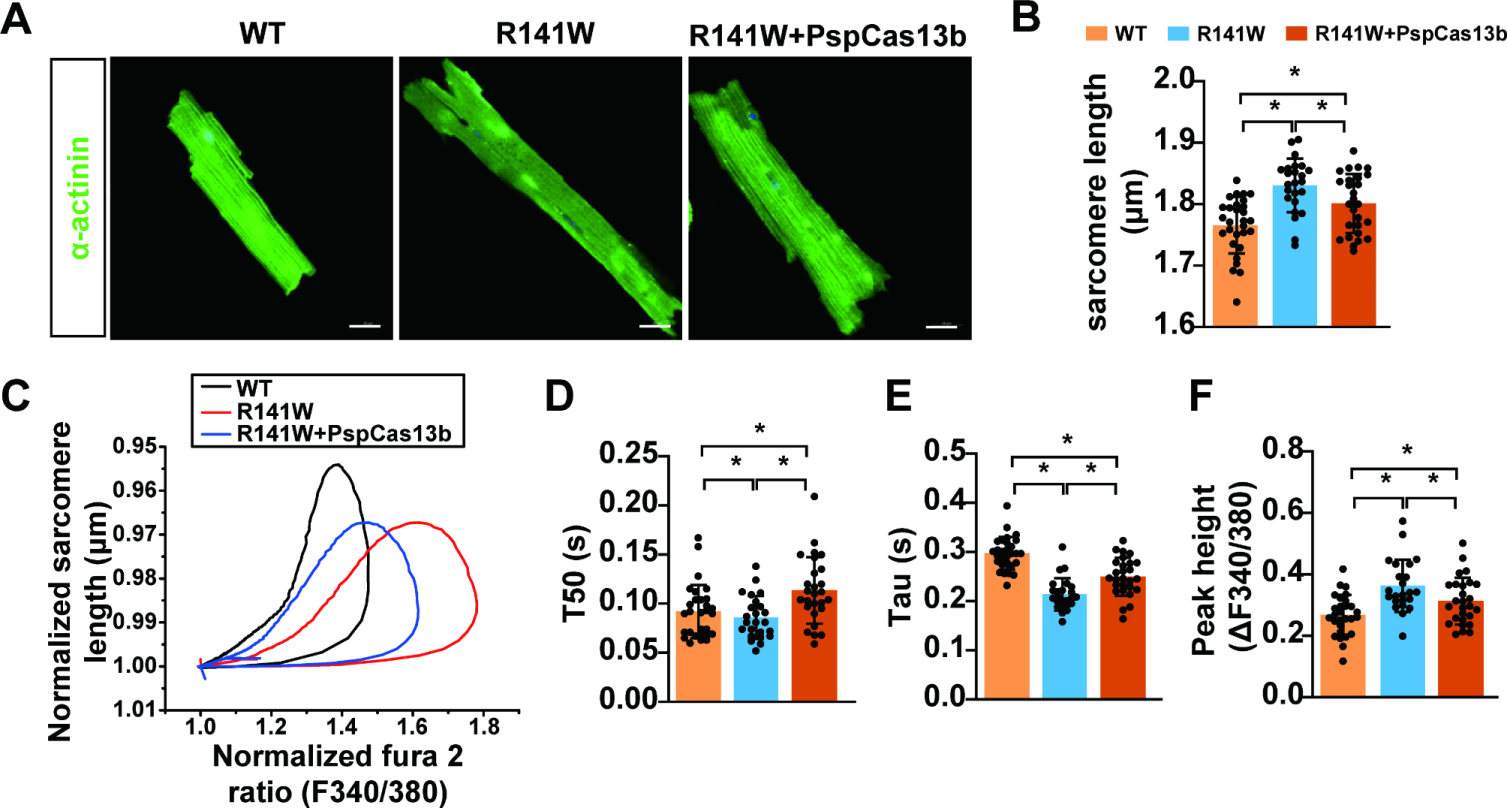
*hTNNT2*^*R141W*^ knockdown by PspCas13b significantly increases myofilament sensitivity to Ca^2+^. (**A**) Size of cardiomyocytes in WT, R141W and AAV9-PspCas13b-treated R141W (R141W + PspCas13b) mice. (**B**) Sarcomere length of cardiomyocytes in WT, R141W and R141W + PspCas13b mice. (**C**) Mean trajectory of normalized sarcomere length vs. fluorescence (F340/380) recorded in WT (n = 28), R141W (n = 21) and R141W + PspCas13b mouse myocytes (n = 26) stimulated at 1 Hz. (**D**) Measurements of T50 (**E**) Tau, and (**F**) peak height; n = 23 to 29 cells per group from 3 mice. Sarcomere and Ca^2+^ transients were recorded at a 1 Hz pacing stimulation frequency with MyoPacer Field Stimulator (IonOptix MA, USA). The error bars represent the SEM. *p < 0.05, **p < 0.01, ***p < 0.001 by one-way ANOVA. T50, time to 50% decay

Reportedly, the p.R141W mutation in *hTNNT2* causes DCM by decreasing Ca^2+^ sensitivity through increased affinity for tropomyosin [35]. To assess the functional improvement mediated by *hTNNT2*^*R141W*^ knockdown on cardiomyocyte contractile function, we measured Ca^2+^ dynamics and contractile properties in PspCas13b-mediated *hTNNT2*^*R141W*^ knockdown cardiomyocytes and compared them with those in WT and untreated R141W mouse cardiomyocytes. The results showed that the size of R141W mouse cardiomyocytes significantly decreased after injection of the AAV9-PspCas13b system (Fig. 4A). Additionally, the sarcomere length of cardiomyocytes in WT and AAV9-PspCas13b-injected R141W mice was significantly shorter than that in R141W mice (Fig. 4B).

We observed a significant increase in the gradient of the trajectory in R141W cardiomyocytes compared to WT cardiomyocytes. Additionally, the trajectory gradient in cardiomyocytes from AAV9-PspCas13b-injected R141W mice was significantly lower than that in cardiomyocytes from untreated R141W mice (Fig. 4C, D). The [Ca^2+^]i levels at which the maximum twitch occurred were 1.38 ± 0.04 (F340/380) for WT (n = 28), 1.58 ± 0.03 (F340/380) for R141W (n = 21), and 1.43 ± 0.04 (F340/380) for AAV9-PspCas13b-treated R141W cardiomyocytes (n = 26). Knockdown of *hTNNT2*^*R141W*^ transcripts using PspCas13b significantly alleviated cardiomyocyte dysfunction, leading to an enhanced Ca^2+^ amplitude (Fig. 4F) and accelerated Ca^2+^ decay (Fig. 4D, E). Collectively, these data demonstrate that *hTNNT2*^*R141W*^ knockdown by PspCas13b significantly increases the sensitivity of myofilaments to Ca^2+^.

### **PspCas13b-mediated*hTNNT2***^***R141W***^**knockdown restores cardiac function**

**Fig. 5:**
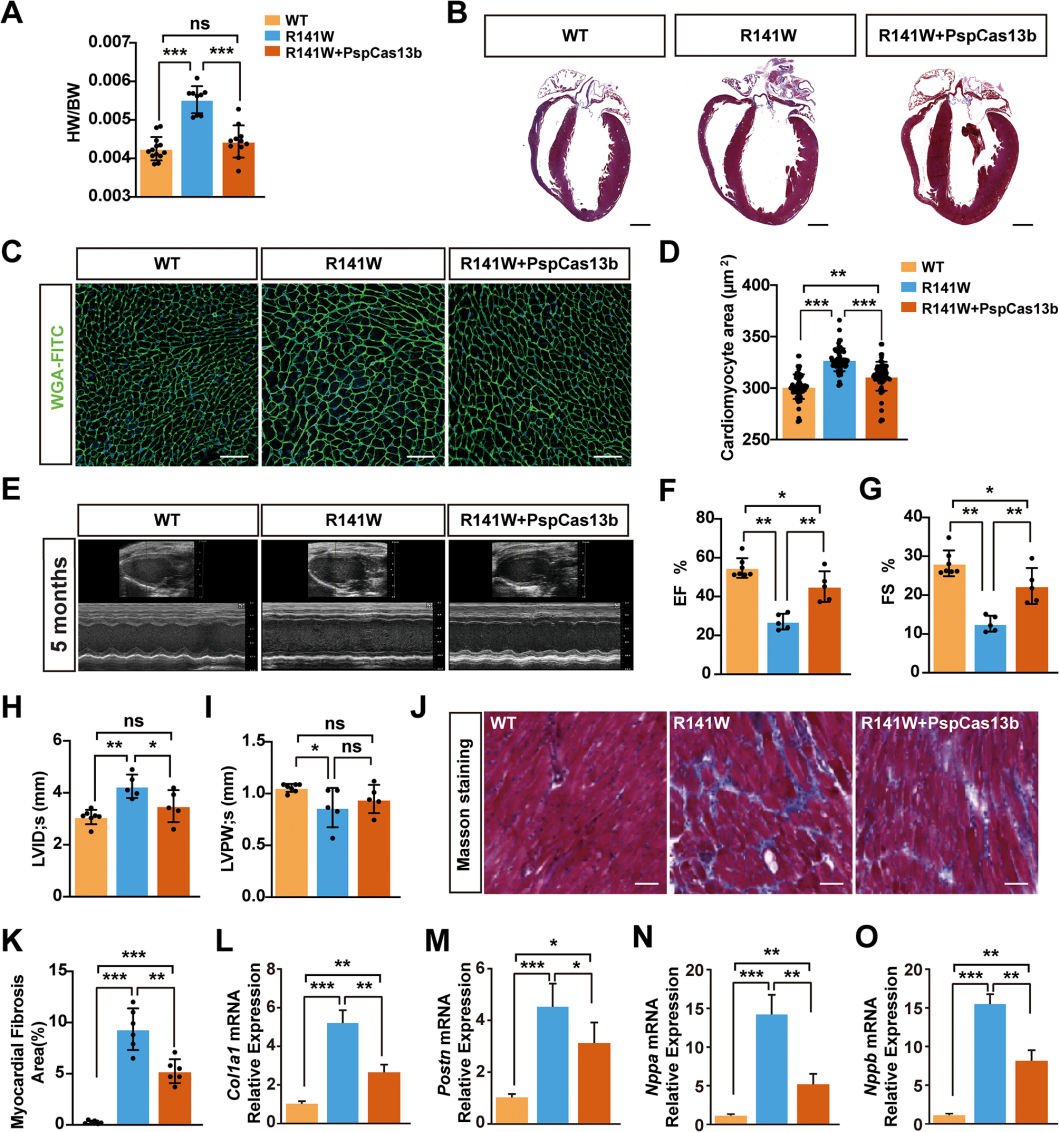
Restoration of cardiac function in DCM through PspCas13b-mediated *hTNNT2*^*R141W*^ knockdown. (**A**) HW/BW ratios of the WT (n = 13), R141W (n = 9) and R141W + PspCas13b (n = 11) groups of mice. The bar graphs show the mean ± SEM. *p < 0.05, **p < 0.01 according to Student’s t test. (**B**) Gross morphology of hearts from 5-month-old WT, R141W, and R141W mice injected with AAV9-PspCas13b-sgRNA2 for 4 weeks. (Scale bars = 500 μm). (**C-D**) FITC–WGA staining of cardiomyocyte membranes from WT, R141W and R141W + PspCas13b mice (n = 68 cardiomyocytes/group). Scale bar = 100 μm. The bar graphs show the mean ± SEM. ***p < 0.001 according to Student’s t test. (**E-I**) Left ventricular performance was measured in WT (n = 7), R141W (n = 5) and R141W + PspCas13b (n = 5) groups of mice 4 weeks after the second tail vein injection. EF, left ventricular ejection fraction (**F**); FS, left ventricular fractional shortening (**G**); LVID;s, systolic left ventricular internal diameter (**H**); LVPW;s, systolic left ventricular posterior wall thickness (**I**). (**J, K**) Representative Masson staining and quantitation of fibrosis in WT, R141W and R141W + PspCas13b mouse hearts (n = 6 mice/group). Scale bar = 50 μm. (**L, M**) Quantification of the relative expression of the fibrosis-related genes collagen type I (*Col1a1*) and periostin (*Postn*) in hearts from WT, R141W and R141W + PspCas13b mice aged 5 months by qRT-PCR using the 2^*-∆∆Ct*^ method (n = 3). The loading was normalized to β-actin. (**N, O**) Quantification of the relative expression of the heart failure genes natriuretic peptide type A (*Nppa*) and natriuretic peptide type B (*Nppb*) in hearts from WT, R141W and R141W + PspCas13b mice aged 5 months by qRT-PCR using the 2^-∆∆Ct^ method (n = 3). The loading was normalized to β-actin. The bar graphs show the mean ± SEM. *p < 0.05, **p < 0.01, ***p < 0.001 by one-way ANOVA [32–34]

To evaluate the therapeutic effects of PspCas13b-mediated knockdown of *hTNNT2*^*R141W*^ in R141W DCM mice, we administered AAV9 vectors containing PspCas13b and sg*TNNT2* through tail vein injection to 4-month-old R141W mice (Fig. 3A). Histological characteristics and cardiac function were assessed in three groups (WT, R141W, and R141W + PspCas13b) four weeks after injection. The *hTNNT2*^*R141W*^ mutation in cardiomyocytes can result in an increased heart-to-body-weight (HW/BW) ratio and increased cardiomyocyte area, both of which are indicators of DCM. After treatment with PspCas13b, we observed a reduction in heart weight in R141W mice (Fig. 5A, B). Wheat germ agglutinin (WGA) staining showed a decrease in cardiomyocyte area in PspCas13b-treated R141W mice (Fig. 5C, D). Echocardiography revealed that R141W mice significantly reduced ejection fraction (EF), fractional shortening (FS), and systolic left ventricular posterior wall thickness (LVPW;s), while the systolic left ventricular internal diameter (LVID;s) was significantly increased compared to that of the WT group (Fig. 5E-I). Following knockdown of *hTNNT2*^*R141W*^ mRNA expression with PspCas13b for four weeks, EF, FS and LVID;s were significantly restored (Fig. 5F-H).

Masson staining revealed that cardiac interstitial collagen content increased in R141W mice, while PspCas13b treatment significantly reduced interstitial collagen deposition in R141W mouse myocardium (Fig. 5J, K). qRT-PCR demonstrated decreased mRNA levels of collagen type I (*Col1a1*) and periostin (*Postn*), the mRNA levels of fibrosis markers, in the myocardium of R141W + PspCas13b mice compared with R141W mice (Fig. 5L, M), indicating the efficacy of PspCas13b treatment in alleviating cardiac interstitial fibrosis in R141W mice. In addition, the mRNA levels of heart failure markers, natriuretic peptide type A (*Nppa*) and natriuretic peptide type B (*Nppb*), which were increased in R141W mice, were sharply decreased after the PspCas13b treatment (Fig. 5N, O). Our findings suggest that CRISPR-Cas13b-mediated knockdown of *hTNNT2*^*R141W*^ mRNA expression can restore cardiac function, reduce cardiac interstitial fibrosis, and ameliorate heart failure in DCM mice.

## Discussion

Cas13b is a CRISPR-associated enzyme known for its high cleavage activity and targeting specificity, which make it an excellent candidate for therapeutic applications. In this study, based on the R141W DCM mouse model, we used the CRISPR-Cas13b system to knock down the mRNA of *hTNNT2*^*R141W*^. Our results demonstrated that this approach restored cardiac function in R141W DCM mice, highlighting the potential of Cas13b as a promising tool for treating this debilitating condition.

In recent years, CRISPR systems have been explored for the treatment of genetic diseases and DNA virus replication due to their efficient DNA editing capabilities [36–39]. However, the potential risks associated with permanent DNA alterations resulting from the standard CRISPR‒Cas9 editing system have limited its clinical application [14–16, 18, 40]. In vivo gene therapy holds great promise for the treatment of cardiovascular diseases, including DCM [41]. RNAi-based approaches, such as siRNA and shRNA treatment, have shown efficacy in improving cardiac function and reducing myocardial fibrosis in animal models of DCM [1, 13, 42]. However, the off-target effects associated with RNAi-based therapies limit their clinical application. In contrast, RNA-targeting CRISPR systems, such as the CRISPR-Cas13b system, offer a promising alternative with potentially lower risks for clinical use.

CRISPR-Cas13b system, which requires single guide RNA to target the intended sequence, was first reported by Feng Zhang in 2017 [18]. CRISPR-Cas13b system has demonstrated a consistent knockdown efficiency of > 80% for the expression of various endogenous mRNAs and lncRNAs [15, 18], with markedly higher specificity than that of spacer-matching shRNAs, and no detectable off-target effects in comparison to the hundreds observed for RNAi. These characteristics position Cas13b as a potentially transformative therapeutic tool for the treatment of diseases marked by abnormal gene expression patterns. The CRISPR-Cas13b system is modifiable to enhance viral RNA clearance, suppress viral replication, and circumvent its mutational escape in order to achieve the goal of controlling viral infections [43–46]. In the treatment of cancer, it is possible to engineer the CRISPR-Casl3b system to regulate cellular methylation, which in turn inhibits tumor progression and controls tumor metastasis [47, 48]. Rashnonejad et al. proposed that *DUX4* silencing is the most direct route to Facioscapulohumeral muscular dystrophy (FSHD) therapy and they developed an AAV6-CRISPR-Cas13b strategy to silence *DUX4* mRNA. Intramuscular delivery of an AAV6 vector encoding a PspCas13b enzyme and *DUX4*-targeting guide RNAs reduced *DUX4* mRNA by > 50% and improved histopathological outcomes in FSHD mice [49]. As of now, there are no reported applications of the CRISPR-Cas13b system in the treatment of cardiovascular diseases. Our study utilized AAV9 delivery to demonstrate that the CRISPR-Cas13b system can effectively cleave targeted mRNA in vivo, knocking down the *hTNNT2*^*R141W*^ mRNA expression and subsequently alleviating genetic DCM in mice. This marks a groundbreaking advancement, as this is the first report of the application of the Cas13b system in treating cardiomyopathy or even genetic cardiovascular disorders to our knowledge, highlighting the potential of Cas13b-mediated gene therapy for cardiomyopathies and potentially genetic diseases that require downregulation of aberrant gene products.

## Conclusions

Overall, our findings indicate that AAV9-mediated administration of PspCas13b can effectively suppress *hTNNT2*^*R141W*^ mRNA expression and improve cardiac function in a murine model of DCM. These results support the potential application of the RNA-targeting CRISPR-Cas13b system as a promising therapeutic strategy for this disorder.

## Materials and methods

### Animals

The α-MHC-*hTNNT2*^*R141W*^ DCM transgenic mice (referred to as R141W mice) were generated previously in the Laboratory of Animal Science of Peking Union Medical College [26]. All of the mice used in this study were maintained on a C57BL/6J genetic background, and all animal experimental procedures were approved by the Ethics Committee for Animal Research of Fuwai Hospital and adhered to the Guide for the Care and Use of Laboratory Animals (NIH Publication No. 85 − 23, revised 1996). Mice were genotyped by PCR with the forward primer 5’-GAACAGGAGGAAGGCTGAGGATGAG-3’ and the reverse primer 5’-TATTTCCAGCGCCCGGTGACTTTAG-3’. The *hTNNT2*^*R141W*^ cDNA was amplified for restriction endonuclease analysis with the forward primer 5’-TTCATGCCCAACTTGGTGC-3’ and the reverse primer 5’-CTCTCTTCAGCCAGGCGGTTC-3’. Restriction endonuclease HpaII was obtained from New England Biolabs, and the digestion was conducted at 37 ℃ for 3 h and separated by electrophoresis. The *hTNNT2*^*R141W*^ cDNA amplification product can be cut into 3 fragments of 147 bp and 66 bp + 44 bp sizes, while the wild-type cDNA cannot be digested and remains unchanged as a product of 260 bp.

### Cell line

The human embryonic kidney 293 (HEK-293T) cell line and human AC16 cardiomyocytes were obtained from the China Infrastructure of Cell Line Resource (NICR) and cultured in DMEM with 10% FBS and 1% penicillin/streptomycin in a 37 °C incubator under 5% CO_2_.

### In vitro sgRNA testing

In accordance with Zhang et al.’s study [15], we have designed 10 sgRNAs specifically targeting the mutation site. The sequences of these sgRNAs are listed in Table 1. To facilitate their binding, we have added a CACC sticky end to the 5’ end of the designed sgRNA sequences and a CAAC sticky end to the 5’ end of the reverse complementary sequences of the sgRNAs. Subsequently, we synthesized 10 pairs of sgRNA oligos, which were further diluted to 100 pM. In the reaction system utilizing T4 PNK (NEB # M0201V), one pair of sgRNA oligos underwent a gradient annealing process from 95 °C to 25 °C, resulting in the formation of dsDNA with double sticky ends. The obtained product was then diluted 1:250 for subsequent applications.

The sgRNA vector (pC0043-PspCas13b crRNA backbone, Addgene #103,854) was enzymatically digested using the BbsI restriction enzyme, and the resulting digested products were recovered utilizing the TIANgel purification kit (TIANGEN #DP219). Following the recovery, the digested sgRNA vector was ligated with the annealed sgRNA using T4 ligase, which proceeded overnight at 16 °C. For the subsequent transfection step, 2400 ng of constructed sgRNA plasmid and 1200 ng of PspCas13b plasmid (pC0046-EF1a-PspCas13b-NES-HIV, Addgene #103,862) were cotransfected into *TNNT2*^*R141W*^ 293T cells in a 12-well plate. These cells had previously undergone infection with the lenti-*TNNT2*^*R141W*^-Puro lentivirus and were selected using puromycin. Following the 48-hour transfection period, total RNA was extracted using the Trizol method, and cDNA was synthesized for subsequent qRT-PCR analysis to assess knockdown efficiency.

**Table 1:** The sequences of sgRNAs designed for the targeted mutation site

sgRNA Number	sgRNA Sequence (5’ − 3’)
sgRNA1	GCTCCTTCTCCCGCTCATTCCAGATGCGCT
sgRNA2	TCCTTCTCCCGCTCATTCCAGATGCGCTGC
sgRNA3	CTTCTCCCGCTCATTCCAGATGCGCTGCTG
sgRNA4	TCTCCCGCTCATTCCAGATGCGCTGCTGCT
sgRNA5	TCCCGCTCATTCCAGATGCGCTGCTGCTCG
sgRNA6	CCGCTCATTCCAGATGCGCTGCTGCTCGGC
sgRNA7	GCTCATTCCAGATGCGCTGCTGCTCGGCCC
sgRNA8	TCATTCCAGATGCGCTGCTGCTCGGCCCGC
sgRNA9	TCCAGATGCGCTGCTGCTCGGCCCGCTCTG
sgRNA10	CCGCTCCTTCTCCCGCTCATTCCAGATGCG

### Testing of the specificity of the PspCas13b system

We developed two mini-constructs containing a fluorescent reporter gene linked to either WT-*TNNT2* or MT-*TNNT2*^*R141W*^, as shown in Fig. 2E. These constructs were designed based on the human *TNNT2* mRNA sequence and differed in the position of the mutation recognition site. To create heterozygous conditions, we cotransfected the two reporter alleles and PspCas13b-sgRNA2 duplexes into cultured HEK-293 cells. We then conducted qRT-PCR to detect the expression levels of GFP and RFP, which enabled us to assess the effectiveness of the CRISPR-Cas13b system in targeting and suppressing the mutant allele while preserving expression of the WT allele.

The WT AC16 cell line was converted into a mutant cell line with a heterozygous *TNNT2*^*R141W*^ allele using SPRY-CBE4max (Fig. 2D). PspCas13b and sgRNA2 were cotransfected into this cell line, and total *TNNT2* expression levels were measured using qRT-PCR. PCR amplicons containing the R141W mutation site were then cloned into the pBackZero-T Vector (TAKARA, Beijing, China) for sequencing via Sanger sequencing, and the results were analyzed using Chromas.

### Testing of the off-target effects of the PspCas13b system

We cotransfected sg*TNNT2*s and PspCas13b vectors into *TNNT2*^*R141W*^ 293T cells that had been infected with EF1α-*TNNT2*^*R141W*^-puro lentivirus. 48 h after transfection, cells were collected for RNA extraction and subsequent transcriptome-wide RNA-Seq. RNA-seq data was analyzed as previously described [50] and presented as the mean of all repeats. The mRNA sequencing (high-throughput) was performed using the Illumina Genome Analyzer and the adapters were removed using Trimmomatic (v0.36) during sequencing. The Hisat2 (v2.0.0) was used to map qualified reads to the mouse reference genome (mm10) with default parameters. Then, the expression levels of all mapped genes were estimated by Stringtie (v2.0) and the gene expression abundances were indicated by FPKM (fragments per kilobase of transcript per million fragments mapped).

### AAV9 production and tail vein injection

AAV9-EF1α-PspCas13b and AAV9-U6-sg*TNNT2* were produced by transfecting HEK293T cells with polyethylenimine (PEI) at a concentration of 50 µg/mL. Briefly, plasmids encoding the viral vector, packaging proteins, and helper reagents were cotransfected into HEK293T cells using PEI. The viruses were then harvested from the cell culture medium 3–7 days after transfection and purified by ultracentrifugation through a cesium chloride gradient. The virus-containing fractions were collected, dialyzed against phosphate-buffered saline (PBS), and concentrated using Amicon Ultra centrifugal filter units (100 kDa cutoff).

To evaluate the therapeutic efficacy of these viruses in vivo, we performed tail vein injections of 4 × 10^12 AAV9 particles (determined by qRT-PCR) into 4-month-old mice, which enabled efficient systemic delivery to the heart. These optimized procedures have been extensively validated and provide robust and reproducible results for studies utilizing RNA-targeting CRISPR-Cas13b technology.

### RNA extraction and quantitative real-time PCR

Total RNA was extracted from HEK-293T cells or heart tissues using TRIzol reagent (Invitrogen, CA, USA) according to the manufacturer’s instructions. The RNA samples were then reverse-transcribed into cDNA using PrimeScript RT Master Mix (Takara, RR036A). qRT-PCR analysis was performed using PowerUp™ SYBR Green Mastermix (Applied Biosystems, Shanghai, China) on a StepOnePlus Real-Time PCR System (Applied Biosystems). The qRT-PCR cycling conditions consisted of an initial denaturation step at 95 °C for 10 min followed by 40 cycles of denaturation at 95 °C for 15 s and annealing/extension at 60 °C for 1 min. Relative gene expression levels were calculated using the 2-∆∆Ct method, with β-actin used as the internal control for normalization. The primers used in this research are listed in Table 2.

**Table 2:** Primers used in this research for qRT-PCR

Gene	Forword primer (5’ − 3’)	Reverse primer (5’ − 3’)
*hTNNT2*	CTATCTCCTCCAGATGGCCC	ACTCTCTCTCCATCGGGGAT
*Tnnt2*	AGAGGACACCAAACCCAAGC	CTCCCGCTCATTGCGAAT
*Nppb*	GTCAGTCGTTTGGGCTGTA	GCTATGTTTATTATGTTGTGGC
*Nppa*	TCCGATAGATCTGCCCTCTT	CTCCAATCCTGTCAATCCTACC
*Col3a1*	ACTTGGAATTGCAGGGCTAAC	CTGGCTCCTGGTTTTCCACT
*Postn*	TGACATACGCAGAGGACTG	TCAATGACATGGACGACAC
*ACTIN*	GTGACAGCAGTCGGTTGG	GCAATGCTATCACCTCCC
*Actin*	AGAGGACACCAAACCCAAGC	CTCCCGCTCATTGCGAAT

### Histology

Mouse hearts were harvested and fixed in 4% paraformaldehyde at room temperature for 24 h. Subsequently, the hearts were dehydrated through an ethanol and xylene series and finally paraffin-embedded. The paraffin-embedded hearts were sliced continuously into 5-mm-thick sections, with five sections collected from each heart. Standard procedures were used to perform hematoxylin and eosin (H&E) and Masson’s trichrome staining.

### Immunofluorescence assay

Immunofluorescence staining was performed on paraffin-embedded tissue sections to visualize the localization and distribution of specific proteins. Deparaffinization, antigen retrieval, and blocking of nonspecific background staining were performed as previously described [51–54]. The tissue sections were incubated with primary antibodies overnight at 4 °C. After washing three times with PBS, the slides were incubated with a fluorescently labeled secondary antibody appropriate for the primary antibody for 1 h at room temperature. The slides were then washed again three times in PBS, counterstained with 4’,6-diamidino-2-phenylindole (DAPI, Sigma), and mounted with VECTASHIELD mounting medium (Vector Labs, CA, USA). The primary antibody used in this study was anti-WGA conjugated with Alexa Fluor 647 (Invitrogen W32466, 20 mg/mL). The secondary antibody used was donkey anti-rabbit conjugated with Alexa Fluor 488 (Invitrogen A-21,206, 1:500 dilution). Fluorescence images were obtained using a ZEISS LSM800 confocal laser scanning microscope, and acquisition settings were kept consistent between samples. All analyses were performed while blinded to the treatment groups, and representative images were chosen to demonstrate key findings.

### Assessment of myofilament sensitivity to Ca^2+^

Single ventricular myocytes were isolated using a modified version of a previously described technique [55]. Animals were euthanized and their hearts were rapidly removed and mounted in Langendorff mode. The hearts were perfused with HEPES-based salt solution (isolation solution) at a physiological temperature (36–37 °C) and a constant flow rate adjusted according to heart weight. The perfusate contained 0.75 mmol/L Ca^2+^. After stabilization, perfusion was continued for 4 min with Ca^2+^-free isolation solution containing 0.1 mM EGTA followed by 6 min with isolation solution containing 0.05 mmol/L Ca^2+^, 0.75 mg/mL collagenase (type 1; Worthington Biochemical Corp., USA), and 0.075 mg/mL protease (type X1V; Sigma, Germany). The ventricles were then excised from the heart, minced, and gently shaken in collagenase-containing isolation solution supplemented with 1% bovine serum albumin (BSA). Cells were filtered from the solution at 4-minute intervals and resuspended in isolation solution containing 0.75 mmol/L Ca^2+^. Ventricular myocytes were isolated from five control and five mutant mice.

### Echocardiography

Left ventricular systolic function was analyzed by echocardiography one month after AAV9 injection using a digital ultrasound system (Vevo2100 Imaging System, Visual Sonics, Toronto, Canada). The conventional left ventricle functional parameter measurements included EF, FS, end-diastolic diameter, and end-systolic diameter.

### Cell surface area measurement

The cell surface area of WGA-stained cells was measured by ImageJ. Approximately 100–200 cardiomyocytes in 20–50 fields of each heart tissue were examined in each experiment.

### Statistical analysis

The data are presented as the mean ± standard error of the mean (SEM). Student’s t test was used to analyze the results of the control and experimental samples. The results with a *p* value ＜ 0.05 were considered statistically significant.

## Data Availability

The datasets used and/or analyzed during the current study are available from the corresponding author upon reasonable request.

## References

[1] Bongianino R, Denegri M, Mazzanti A, Lodola F, Vollero A, Boncompagni S (2017). Allele-specific silencing of mutant mRNA rescues ultrastructural and arrhythmic phenotype in mice carriers of the R4496C mutation in the ryanodine receptor gene (RYR2). Circul Res.

[2] Koeppen M, Lee JW, Seo SW, Brodsky KS, Kreth S, Yang IV (2018). Hypoxia-inducible factor 2-alpha-dependent induction of amphiregulin dampens myocardial ischemia-reperfusion injury. Nat Commun.

[3] Kumar VB, Medhi H, Yong Z, Paik P (2016). Designing idiosyncratic hmPCL-siRNA nanoformulated capsules for silencing and cancer therapy. Nanomedicine: Nanatechnol Biology Med.

[4] Ozpolat B, Sood AK, Lopez-Berestein G (2014). Liposomal siRNA nanocarriers for cancer therapy. Adv Drug Deliv Rev.

[5] Sarett SM, Werfel TA, Lee L, Jackson MA, Kilchrist KV, Brantley-Sieders D (2017). Lipophilic siRNA targets albumin in situ and promotes bioavailability, Tumor penetration, and carrier-free gene silencing. Proc Natl Acad Sci USA.

[6] Alterman JF, Godinho B, Hassler MR, Ferguson CM, Echeverria D, Sapp E (2019). A divalent siRNA chemical scaffold for potent and sustained modulation of gene expression throughout the central nervous system. Nat Biotechnol.

[7] Wang P, Zheng X, Guo Q, Yang P, Pang X, Qian K (2018). Systemic delivery of BACE1 siRNA through neuron-targeted nanocomplexes for treatment of Alzheimer’s Disease. J Controlled Release: Official J Controlled Release Soc.

[8] Zheng M, Tao W, Zou Y, Farokhzad OC, Shi B (2018). Nanotechnology-based strategies for siRNA brain delivery for Disease Therapy. Trends Biotechnol.

[9] Kim HS, Son YJ, Yoo HS (2016). Clustering siRNA conjugates for MMP-responsive therapeutics in chronic wounds of diabetic animals. Nanoscale.

[10] Li N, Luo HC, Ren M, Zhang LM, Wang W, Pan CL (2017). Efficiency and safety of β-CD-(D(3))(7) as siRNA carrier for decreasing Matrix Metalloproteinase-9 expression and improving Wound Healing in Diabetic rats. ACS Appl Mater Interfaces.

[11] Wen D, Peng Y, Liu D, Weizmann Y, Mahato RI (2016). Mesenchymal stem cell and derived exosome as small RNA carrier and immunomodulator to improve islet transplantation. J Controlled Release: Official J Controlled Release Soc.

[12] Evers B, Jastrzebski K, Heijmans JP, Grernrum W, Beijersbergen RL, Bernards R (2016). CRISPR knockout screening outperforms shRNA and CRISPRi in identifying essential genes. Nat Biotechnol.

[13] Rao DD, Vorhies JS, Senzer N, Nemunaitis J (2009). siRNA vs. shRNA: similarities and differences. Adv Drug Deliv Rev.

[14] Abudayyeh OO, Gootenberg JS, Essletzbichler P, Han S, Joung J, Belanto JJ (2017). RNA targeting with CRISPR-Cas13. Nature.

[15] Cox DBT, Gootenberg JS, Abudayyeh OO, Franklin B, Kellner MJ, Joung J (2017). RNA editing with CRISPR-Cas13. Science.

[16] Gootenberg JS, Abudayyeh OO, Lee JW, Essletzbichler P, Dy AJ, Joung J (2017). Nucleic acid detection with CRISPR-Cas13a/C2c2. Science.

[17] Li J, Zhu D, Hu S, Nie Y (2022). CRISPR-CasRx knock-in mice for RNA degradation. Sci China Life Sci.

[18] Smargon AA, Cox DBT, Pyzocha NK, Zheng K, Slaymaker IM, Gootenberg JS (2017). Cas13b is a type VI-B CRISPR-Associated RNA-Guided RNase differentially regulated by Accessory proteins Csx27 and Csx28. Mol Cell.

[19] McKenna WJ, Maron BJ, Thiene G, Classification (2017). Epidemiology, and Global Burden of Cardiomyopathies. Circul Res.

[20] Richardson P, McKenna W, Bristow M, Maisch B, Mautner B, O’Connell J (1996). Report of the 1995 World Health Organization/International Society and Federation of Cardiology Task Force on the definition and classification of cardiomyopathies. Circulation.

[21] Lian H, Song S, Chen W, Shi A, Jiang H, Hu S (2023). Genetic characterization of dilated cardiomyopathy patients undergoing heart transplantation in the Chinese population by whole-exome sequencing. J Transl Med.

[22] Favalli V, Serio A, Grasso M, Arbustini E (2016). Genetic causes of dilated cardiomyopathy. Heart.

[23] Garcia-Pavia P, Cobo-Marcos M, Guzzo-Merello G, Gomez-Bueno M, Bornstein B, Lara-Pezzi E (2013). Genetics in dilated cardiomyopathy. Biomark Med.

[24] Li D, Czernuszewicz GZ, Gonzalez O, Tapscott T, Karibe A, Durand JB (2001). Novel cardiac troponin T mutation as a cause of familial dilated cardiomyopathy. Circulation.

[25] Yin Z, Ren J, Guo W (2015). Sarcomeric protein isoform transitions in cardiac muscle: a journey to Heart Failure. Biochim Biophys Acta.

[26] Juan F, Wei D, Xiongzhi Q, Ran D, Chunmei M, Lan H (2008). The changes of the cardiac structure and function in cTnTR141W transgenic mice. Int J Cardiol.

[27] Lu D, Ma Y, Zhang W, Bao D, Dong W, Lian H (2012). Knockdown of cytochrome P450 2E1 inhibits oxidative stress and apoptosis in the cTnT(R141W) dilated cardiomyopathy transgenic mice. Hypertension.

[28] Zhao HP, Lu D, Zhang W, Zhang L, Wang SM, Ma CM (2010). Protective action of tetramethylpyrazine phosphate against dilated cardiomyopathy in cTnT(R141W) transgenic mice. Acta Pharmacol Sin.

[29] Zhang W, Lu D, Dong W, Zhang L, Zhang X, Quan X (2011). Expression of CYP2E1 increases oxidative stress and induces apoptosis of cardiomyocytes in transgenic mice. FEBS J.

[30] Lu D, Bao D, Dong W, Liu N, Zhang X, Gao S (2016). Dkk3 prevents familial dilated cardiomyopathy development through wnt pathway. Lab Invest.

[31] Ahmad F, Banerjee SK, Lage ML, Huang XN, Smith SH, Saba S (2008). The role of cardiac troponin T quantity and function in cardiac development and dilated cardiomyopathy. PLoS ONE.

[32] Cardoso AC, Lam NT, Savla JJ, et al. Mitochondrial substrate utilization regulates cardiomyocyte cell cycle progression. Nat Metab. 2020;2(2):167–78. PMC7331943 32617517

[33] Reuter SP, Soonpaa MH, Field D, et al. Cardiac troponin I-interacting kinase affects cardiomyocyte S-phase activity but not cardiomyocyte proliferation. Circulation. 2023;147(2):142–53. 10.1161/CIRCULATIONAHA.122.061130 PMC9839600 36382596

[34] Morikawa Y, Heallen T, Leach J, Xiao Y, Martin JF. Dystrophin-glycoprotein complex sequesters Yap to inhibit cardiomyocyte proliferation. Nature. 2017;547(7662):227–31. 10.1038/nature22979 PMC5528853 28581498

[35] Lu QW, Morimoto S, Harada K, Du CK, Takahashi-Yanaga F, Miwa Y (2003). Cardiac troponin T mutation R141W found in dilated cardiomyopathy stabilizes the troponin T-tropomyosin interaction and causes a Ca2 + desensitization. J Mol Cell Cardiol.

[36] Liu C, Zhang L, Liu H, Cheng K (2017). Delivery strategies of the CRISPR-Cas9 gene-editing system for therapeutic applications. J Controlled Release: Official J Controlled Release Soc.

[37] Mout R, Ray M, Lee YW, Scaletti F, Rotello VM (2017). Vivo delivery of CRISPR/Cas9 for therapeutic gene editing: Progress and challenges. Bioconjug Chem.

[38] Matre PR, Mu X, Wu J, Danila D, Hall MA, Kolonin MG (2019). CRISPR/Cas9-Based Dystrophin Restoration reveals a Novel Role for Dystrophin in Bioenergetics and stress resistance of muscle progenitors. Stem cells (Dayton. Ohio).

[39] Amoasii L, Li H, Zhang Y, Min YL, Sanchez-Ortiz E, Shelton JM (2019). In vivo non-invasive monitoring of dystrophin correction in a new Duchenne muscular dystrophy reporter mouse. Nat Commun.

[40] Crooke ST, Witztum JL, Bennett CF, Baker BF (2018). RNA-Targeted Ther Cell Metabolism.

[41] Pang XF, Lin X, Du JJ, Zeng DY (2020). LTBP2 knockdown by siRNA reverses myocardial oxidative stress injury, fibrosis and remodelling during dilated cardiomyopathy. Acta Physiologica (Oxford England).

[42] Gomes MJ, Martins S, Sarmento B (2015). siRNA as a tool to improve the treatment of brain Diseases: mechanism, targets and delivery. Ageing Res Rev.

[43] Cui J, Techakriengkrai N, Nedumpun T, Suradhat S (2020). Abrogation of PRRSV infectivity by CRISPR-Cas13b-mediated viral RNA cleavage in mammalian cells. Sci Rep.

[44] Yu D, Han HJ, Yu J, Kim J, Lee GH, Yang JH (2023). Pseudoknot-targeting Cas13b combats SARS-CoV-2 Infection by suppressing viral replication. Mol Ther.

[45] Fareh M, Zhao W, Hu W, Casan JML, Kumar A, Symons J (2021). Reprogrammed CRISPR-Cas13b suppresses SARS-CoV-2 replication and circumvents its mutational Escape through mismatch tolerance. Nat Commun.

[46] Chen P, Chen M, Chen Y, Jing X, Zhang N, Zhou X (2022). Targeted inhibition of Zika virus Infection in human cells by CRISPR-Cas13b. Virus Res.

[47] Su G, Yao L, Han X, Liao X, Chen J, Yin T (2022). A synthetic targeted RNA demethylation system based on CRISPR-Cas13b inhibits Bladder cancer progression. Clin Transl Med.

[48] Shi HZ, Xiong JS, Gan L, Zhang Y, Zhang CC, Kong YQ (2022). N6-methyladenosine reader YTHDF3 regulates Melanoma Metastasis via its ‘executor’LOXL3. Clin Transl Med.

[49] Rashnonejad A, Amini-Chermahini G, Taylor N, Fowler A, Kraus E, King O (2022). FP.29 AAV-CRISPR-Cas13 gene therapy for FSHD: DUX4 gene silencing efficacy and immune responses to Cas13b protein. Neuromuscul Disord.

[50] Zhou H, Su J, Hu X, Zhou C, Li H, Chen Z, et al. Glia-to-neuron conversion by CRISPR-CasRx alleviates symptoms of neurological disease in mice. Cell. 2020;181(3):590–603.e16. 10.1016/j.cell.2020.03.024 32272060

[51] Han C, Nie Y, Lian H, Liu R, He F, Huang H (2015). Acute inflammation stimulates a regenerative response in the neonatal mouse heart. Cell Res.

[52] Li Y, Feng J, Li Y, Hu S, Nie Y (2020). Achieving stable myocardial regeneration after apical resection in neonatal mice. J Cell Mol Med.

[53] Yue Z, Chen J, Lian H, Pei J, Li Y, Chen X (2019). PDGFR-β signaling regulates cardiomyocyte proliferation and myocardial regeneration. Cell Rep.

[54] Wang Y, Li Y, Feng J, Liu W, Li Y, Liu J (2020). Mydgf promotes Cardiomyocyte proliferation and neonatal heart regeneration. Theranostics.

[55] Feng J, Li Y, Nie Y (2022). Methods of mouse cardiomyocyte isolation from postnatal heart. J Mol Cell Cardiol.

